# Supplementary material for: Nomogram-Based Prediction of Survival in Stage IV Nasopharyngeal Carcinoma: A Retrospective Single-Center Study
Source: Diagnostics (Basel). 2025 May 23;15(11):1309. doi: 10.3390/diagnostics15111309 (PMC12154514; doi:10.3390/diagnostics15111309)
Supplement: Supplementary file 1 [file diagnostics-15-01309-s001.zip › Table S1.pdf]

Table S1. Hemogram of stage IV nasopharyngeal carcinoma patients.

	Before treatment (Mean±SD)	After treatment (Mean±SD)	Delta (Mean±SD)
Hemoglobulin (gm/dl)	14.01±1.92(8.6-18.5)	11.39±1.65(7.5-14.6)	-2.62±1.88(-8.1-2.4)
LMR	5.26±5.89(1.63-38.50)	2.35±1.52(0.5-10.71)	-2.90±5.89(-35.29-1.60)
PLR	1.69±0.78(0.62-4.36)	3.06±1.68(0.55-9.30)	1.37±1.66(-1.88-6.55)
NLR	3.20±1.58(0.97-6.69)	4.80±3.13(0.2-21.25)	1.60±3.18(-3.14-16.76)
SII	846.99±539.29(121.59-2467.75)	844.61±415.54(4.2-2125)	-2.38±574.37(-1715.43-1229.86)
SIRI	154.41±118.73(11.83-467.80)	169.71±119.49(0.71-802.40)	15.3±157.67(-344.47-505.20)
WBC (10^3/μl)	8.5±3.1 (2.9-20.9)	6.5±3.8 (1.1-25.4)	-2±3.8 (-17.1-13.7)

LMR: lymphocyte/monocyte ratio; PLR: platelet/lymphocyte ratio; NLR: neutrophil/lymphocyte ratio; SII: systemic immune inflammation index; SIRI: systemic inflammation response index
