# Supplementary material for: Nomogram-Based Prediction of Survival in Stage IV Nasopharyngeal Carcinoma: A Retrospective Single-Center Study
Source: Diagnostics (Basel). 2025 May 23;15(11):1309. doi: 10.3390/diagnostics15111309 (PMC12154514; doi:10.3390/diagnostics15111309)
Supplement: Supplementary file 1 [file diagnostics-15-01309-s001.zip › Table S2.pdf]

Table S2. Association of posttreatment BMI, change in BMI, and pretreatment SIRI with disease persistence/recurrence in patients with stage IV nasopharyngeal carcinoma.

		Disease persistence/ recurrence(n=36)	None (n=25)	p-value
Posttreatment BMI (kg/m2)	<21.6	22 (78.57%)	6 (21.43%)	0.004
	≥21.6	14 (42.42%)	19 (57.58%)	
Pretreatment SIRI	1<125	14 (43.75%)	18 (56.25%)	0.011
	≥125	22 (75.86%)	7 (24.14%)	
ΔBMI (kg/m2)	<-1.93	26 (72.22%)	10 (27.78%)	0.012
	≥-1.93	10 (40%)	15 (60%)	
