# Supplementary figures and images for: Nomogram-Based Prediction of Survival in Stage IV Nasopharyngeal Carcinoma: A Retrospective Single-Center Study
Source: Diagnostics (Basel). 2025 May 23;15(11):1309. doi: 10.3390/diagnostics15111309 (PMC12154514; doi:10.3390/diagnostics15111309)

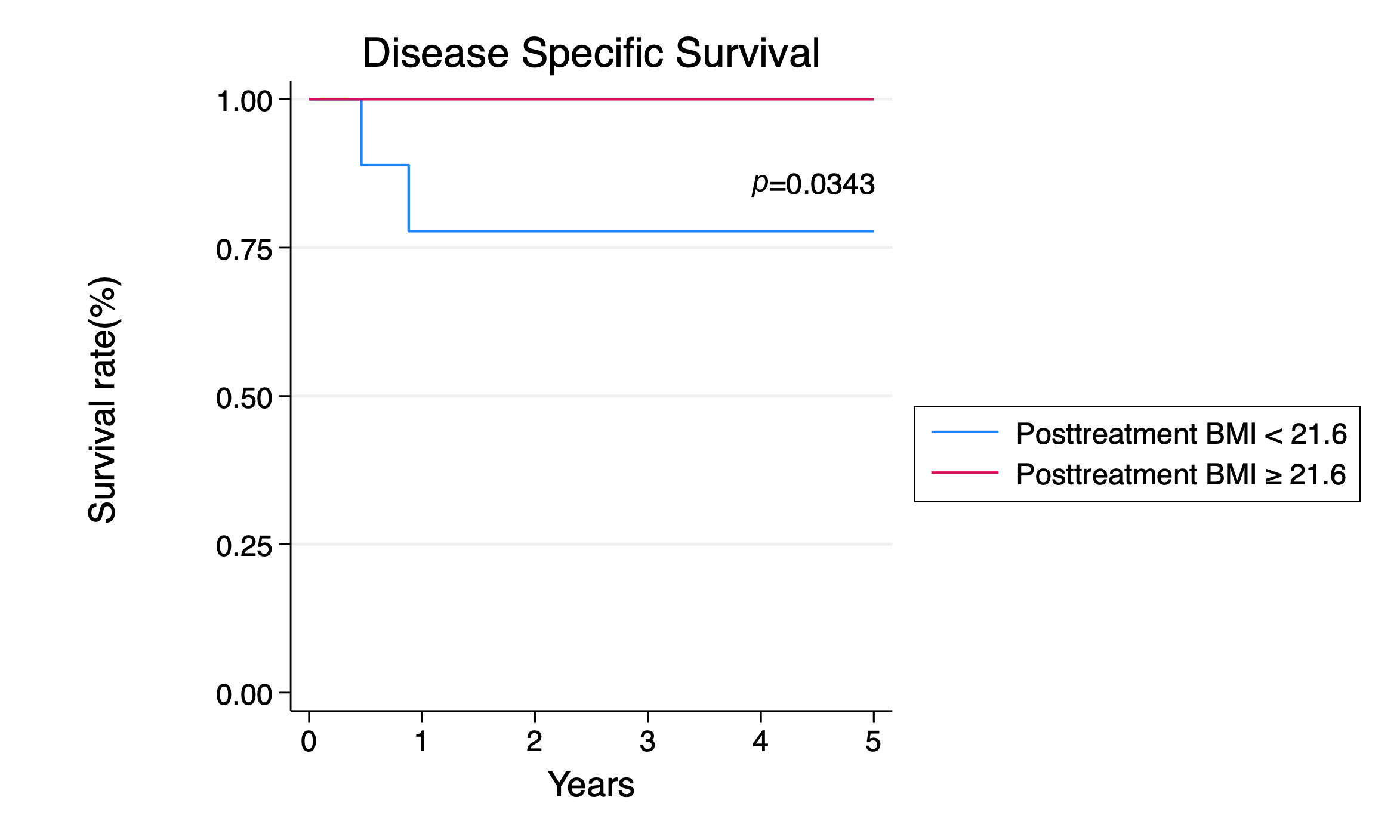

Supplement: Supplementary file 1 [file diagnostics-15-01309-s001.zip › Figure S1.tif]

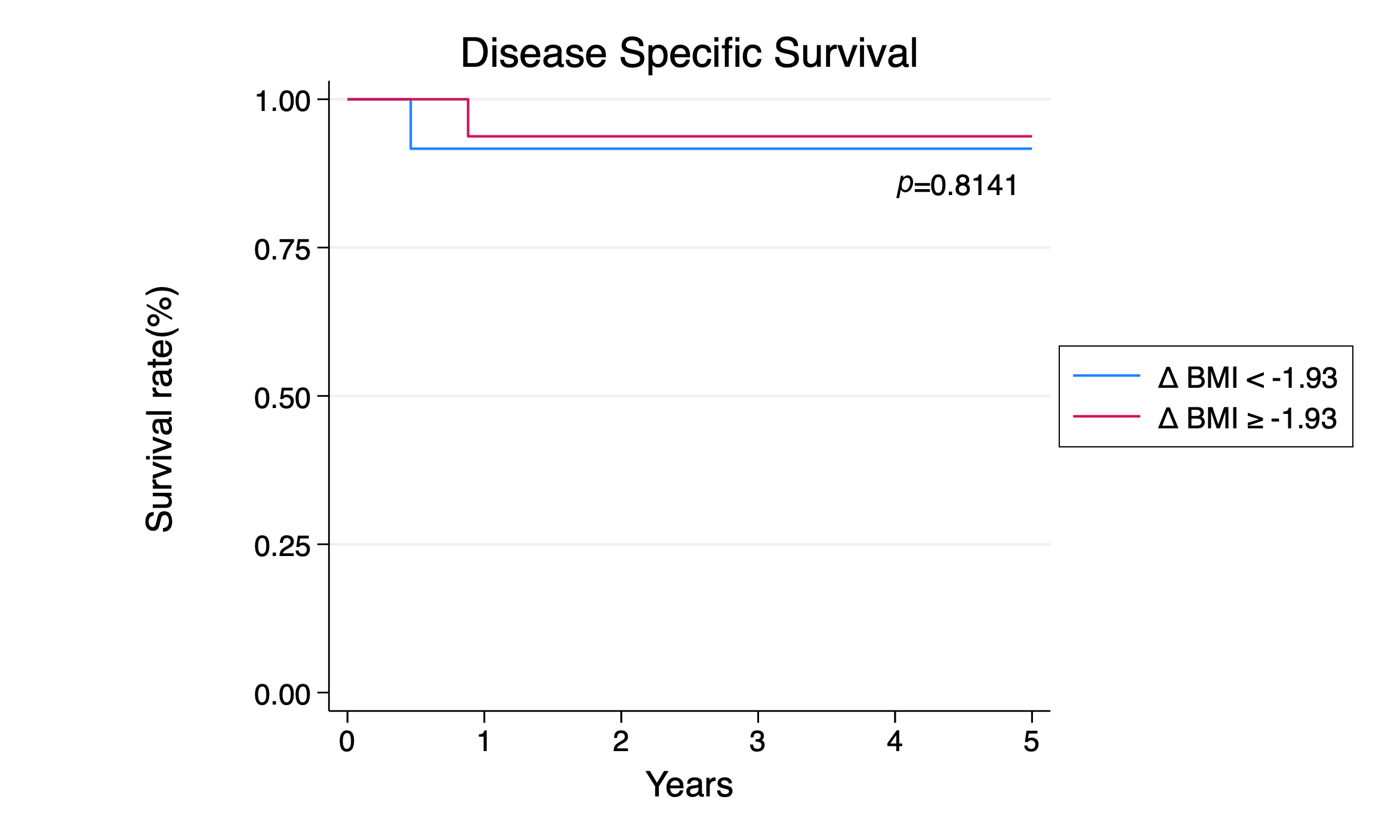

Supplement: Supplementary file 1 [file diagnostics-15-01309-s001.zip › Figure S2.tif]
